# Serum concentrations of gabapentin in cats with chronic kidney disease

**DOI:** 10.1177/1098612X221077017

**Published:** 2022-02-23

**Authors:** Jessica M Quimby, Sarah K Lorbach, Ashlie Saffire, Amanda Kennedy, Luke A Wittenburg, Turi K Aarnes, Karina J Creighton, Sarah E Jones, Rene E Paschall, Emily M King, Clara E Bruner, Jessica N Wallinger, Karen A van Haaften

**Affiliations:** 1 Department of Veterinary Clinical Sciences, College of Veterinary Medicine, The Ohio State University, Columbus, OH, USA; 2 Cats Only Veterinary Clinic, Columbus, OH, USA; 3 Department of Surgical and Radiological Sciences, School of Veterinary Medicine, University of California – Davis, CA, USA; 4 British Columbia SPCA, Vancouver, BC, Canada

**Keywords:** Anxiety, stress, renal failure, half-life, elimination rate

## Abstract

**Objectives:**

The purpose of this study was to assess serum concentrations of gabapentin in cats with chronic kidney disease (CKD) vs clinically healthy cats.

**Methods:**

Five healthy cats were enrolled in a pharmacokinetic study. A single 20 mg/kg dose of gabapentin was administered orally and blood was obtained at 0, 0.25, 0.5, 1, 1.5, 2, 3, 4, 8, 12, 24 and 36 h via a jugular catheter. Serum gabapentin concentrations were measured using liquid chromatography coupled to tandem mass spectrometry. Non-compartmental pharmacokinetic analysis was performed. The same five healthy cats plus 25 cats with stable International Renal Interest Society stage 2 (n = 14) and 3 (n = 11) CKD were enrolled in a limited sampling study. Cats in both groups received a single 10 mg/kg dose of gabapentin, and serum gabapentin concentrations and compliance scores were obtained 3 and 8 h post-administration.

**Results:**

Cats with CKD had significantly higher dose-normalized serum gabapentin concentrations than normal cats at 3 h (*P* = 0.0012 CKD vs normal 10 mg/kg; *P* = 0.008 CKD vs normal 20 mg/kg) and 8 h (*P* <0.0001 CKD vs normal 10 mg/kg; *P* <0.0001 CKD vs normal 20 mg/kg). Both 3 and 8 h dose-normalized serum gabapentin concentrations were significantly correlated with serum creatinine (3 h: *P* = 0.03, *r* = 0.39; 8 h: *P* = 0.001, *r* = 0.57) and symmetric dimethylarginine (3 h: *P* = 0.03, *r* = 0.41; 8 h: *P* = 0.007, *r* = 0.48). There was a significant correlation between 3 h serum gabapentin concentrations and compliance scores (*P* = 0.0002, *r* = 0.68).

**Conclusions and relevance:**

Cats with CKD that received 10 mg/kg of gabapentin had significantly higher dose-normalized serum concentrations than normal cats that received 20 mg/kg, supporting the need to dose-reduce in this patient population.

## Introduction

Stress associated with transportation, examination and diagnostic procedures is a major barrier to the feline patient’s ability to receive veterinary care.^1^ Multiple strategies have been explored to reduce stress and increase compliance for veterinary examinations, including behavioral conditioning,^2^ low-stress handling^3^ and fast-acting anxiolytic medications.^4^ Gabapentin is a medication that, although traditionally prescribed for chronic pain and seizures, has recently become popular to reduce stress and improve compliance during veterinary visits.^5,6^ The results of a placebo-controlled study confirmed the beneficial effect of gabapentin in reducing stress and came to the conclusion that 20 mg/kg was an effective dose for healthy adult cats.^5^

The 20 mg/kg stress-reduction dose of gabapentin may be beneficial to facilitate preventive veterinary care in younger, healthy cats, but this dose may be inappropriate for elderly cats, specifically those with chronic kidney disease (CKD). Gabapentin is not metabolized or protein bound, and is cleared only by renal excretion in humans; it is unknown whether this is also true in cats.^7^ In humans, it has been demonstrated that kidney disease significantly influences the pharmacokinetics (PK) of gabapentin, and a 60% and 85% decrease in gabapentin clearance is seen in moderate and severe renal dysfunction, respectively.^7^ As a result of these alterations, dose reduction in human patients with CKD is recommended to prevent toxicity.^7,8^ Toxicity in human patients with CKD can manifest as reduced consciousness, unsteady gait or ataxia, dizziness or weakness, myoclonus, confusion, tremulousness and asterixis, and is more pronounced in patients with decreased renal function who have received higher doses.^7,8^ In cats, anecdotal experience is similar in that higher gabapentin doses (20 mg/kg or 100 mg/cat) can lead to excessive sedation and hypotension in patients with CKD, and a dose decrease of at least 50% is commonly used in clinical practice.

As a result of these observations, it is necessary to explore the serum concentrations of gabapentin in feline CKD so that these patients could more safely benefit from its use. Although the PK of gabapentin have previously been determined in normal cats,^9,10^ no information exists on the PK of the 20 mg/kg dose used to reduce stress or its potential alteration in CKD. Therefore, the purpose of this study was to characterize the PK of gabapentin administered at the 20 mg/kg stress-reduction dose in normal cats and compare this with the serum concentrations of gabapentin in cats with CKD at a 50% reduced dose of 10 mg/kg. Our hypotheses were that dose-normalized gabapentin serum concentrations would be higher in cats with CKD than in normal cats, and that this would be correlated with disease severity. A secondary hypothesis was that the reduced dose of 10 mg/kg would still result in compliance during manipulation at the 3 h time point (clinically relevant ‘gabapentin window’ for working with patients on gabapentin) vs the 8 h time point in cats with CKD.

## Materials and methods

### Cats

#### Normal cats

Five client-owned healthy cats with normal systemic blood pressure, serum biochemistry profile, symmetric dimethylarginine (SDMA), complete blood count (CBC), urinalysis and serum total thyroxine (TT4) measurement were enrolled. Healthy control cats were defined as those with no clinical abnormalities, normal physical examination, creatinine <1.6 mg/dl, urine specific gravity >1.035 and SDMA <14.

#### Cats with CKD

Cats with stable International Renal Interest Society (IRIS) stage 2 and 3 CKD were enrolled. Diagnostic tests required before enrollment included systemic blood pressure, serum biochemistry profile, CBC, urinalysis and serum TT4 measurement. Cats with CKD were considered to have stable CKD if serum creatinine had not changed by more than 20% on at least two measurements taken at least 2 weeks apart. Exclusion criteria included other uncontrolled systemic illnesses, complications of CKD such as pyelonephritis or ureteral obstruction, decompensation of CKD requiring hospitalization, and intravenous fluid or current gabapentin therapy. Other concurrent therapies for CKD such as dietary management, potassium supplementation, antihypertensive medications and subcutaneous fluids were acceptable.

### Gabapentin preparation

Capsules were compounded in 5 mg increments (eg, 20 mg, 25 mg, 30 mg) by the Ohio State University Veterinary Medical Center Pharmacy following compounding standards set by US Pharmacopeia (USP) chapters 795 and 1163. All of the capsules were filled with the same active pharmaceutical ingredient (API): gabapentin, bulk USP Powder (lot DR0198; Attix Pharma). The ‘excipient’ for all of the capsules was Lactose NF Powder (lot 151894A; Medisca). All of the capsules were filled using a manually operated capsule-filling machine (ProFiller 100; Torpac). The API and excipient were weighed according to the required quantity for each capsule strength and then thoroughly blended before being used to fill empty gelatin capsules. Capsules were prepared in batches of 10 for each desired concentration. Capsules from each batch were weighed after preparation to verify percentage error range. No capsule had >5% error; the average capsule weight was within 99.4% of the projected final weight.

### Gabapentin administration and sample collection

For the PK study in normal cats, a double-lumen jugular catheter was placed under anesthesia 12–16 h before study initiation to facilitate low-stress sample collection. A single dose of 20 mg/kg gabapentin (rounded to the nearest 5 mg) was administered orally followed by 3 ml of water. Blood samples were obtained at 0, 0.25, 0.5, 1, 1.5, 2, 3, 4, 8, 12, 24 and 36 h after the administration of gabapentin. For the limited-sampling study in normal cats and cats with CKD, a single dose of 10 mg/kg gabapentin (rounded to the nearest 5 mg) was administered orally followed by 3 ml of water. For both studies, blood was transferred into red-top tubes, centrifuged immediately after clot formation (1300 *g* for 10 mins in a cooled [4°C] centrifuge), and serum was separated and stored at −80°C.

### Compliance assessment

Compliance scores were obtained at 3 h and 8 h for cats with CKD during the limited-sampling study.^5^ The compliance score (3 = no resistance to handling, 2 = minimally resistant to handling, 1 = struggling, difficult to handle, 0 = extreme struggling with or without urination or defecation) was assigned by the attending clinician for each of three activities: drawing the blood sample; a mock palpation and isolation of bladder (as if for cystocentesis in lateral recumbency); and placement of the cat in dorsal recumbency in a padded ultrasound trough. The compliance scores from the three activities were summed to reflect the fact that higher summed scores represent a more compliant patient.

### Gabapentin serum concentration analysis

Measurement of gabapentin serum concentrations was performed by the Bio-analytical Research Core, Department of Molecular Biosciences, University of California – Davis using a previously published liquid chromatography coupled to tandem mass spectrometry (LC–MS/MS)-based assay for the analysis of gabapentin in cat plasma.^9^ A partial validation was performed using feline serum as a matrix. Briefly, the assay was performed with low and high concentration calibration curves in blank feline serum. The lower limit of quantitation (LoQ) was 2 ng/ml and the low concentration curve was linear between 2 ng/ml and 2000 ng/ml; the high concentration curve was linear between 2000 ng/ml and 30,000 ng/ml (*r*^2^ >0.99, 1 × weighting for both). The accuracy of the calibration samples was within 15% at all concentrations other than the LoQ, where it was 16%. Quality control samples were run at 10 ng/ml and 10,000 ng/ml (n = 6 concentration/assay) and the intra- and inter-day accuracy and precision were within 15% and 10%, respectively.

### Gabapentin capsule analysis

Gabapentin working solutions were prepared by dilution of the stock solution with methanol to concentrations of 10 and 100 ng/µl. Calibrators were prepared by dilution of the working standard solutions with 5% acetonitrile in water, with 0.2% formic acid, to concentrations ranging from 0.05 to 2 ng/µl. Prior to analysis, gabapentin pills (n = 4) were dissolved in 100 ml acetone:water (1:1 v:v) and diluted further 100-fold in acetone:water (1:1 v:v). The samples then underwent a final 10-fold dilution in 5% acetonitrile in water, with 0.2% formic acid and 10 µl was injected in the LC–MS/MS system. The analytical method used for the LC–MS/MS analysis was as described for the blood samples.

### PK analysis

For the PK study in normal cats, non-compartmental PK analysis was performed using a commercially available PK software program (Phoenix WinNonlin v7).

### Statistical analysis

Data were assessed for normality using the D’Agostino and Pearson test, and analyzed as parameteric or non-parametric data accordingly. Spearman rank was used to assess correlation between gabapentin concentration and compliance scores at 3 h and 8 h, and correlation between dose-normalized gabapentin concentration and serum creatinine or SDMA at 3 h and 8 h. Dose-normalized gabapentin serum concentrations at 3 h and 8 h were compared between cats with CKD receiving 10 mg/kg and both normal cats receiving 20 mg/kg and normal cats receiving 10 mg/kg using a Mann–Whitney test. Compliance scores were compared between 3 h and 8 h using the Wilcoxon sign rank test.

## Results

### Cats

Five apparently healthy and 27 cats with CKD completed the study. Two cats with CKD were excluded from analysis because their serum gabapentin concentrations were higher at 8 h than at 3 h. Of the 25 cats with CKD that were included in data analysis, 14 were IRIS stage 2 and 11 were IRIS stage 3. Cats with CKD had a median age of 13 years (range 2–18) and a median serum creatinine 2.6 mg/dl (range 1.6–4.8) and consisted of 17 domestic shorthairs (DSH), four domestic longhairs and one each of domestic mediumhairs, Siamese cross, Himalayan and Maine Coon (13 male castrated and 12 female spayed). Apparently healthy cats had a median age of 1.6 years (range 1–7) and a median serum creatinine 0.9 mg/dl (range 0.9–1.3) and consisted of five DSH (three male castrated and two female spayed).

### Gabapentin capsule analysis

When compounded capsules used in the study were analyzed for gabapentin concentration, the median dose was 92% of target dose (range 90–104%).

### Normal cat PK study

**Figure 1:**
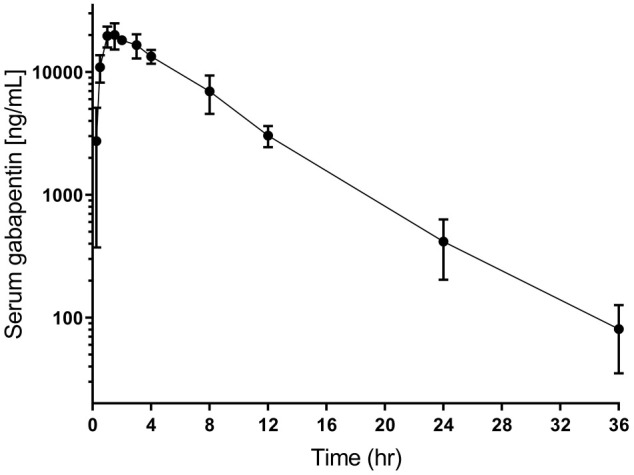
Mean ± SD serum gabapentin concentration over time in five normal cats following oral administration of single dose of 20 mg/kg gabapentin

PK parameters for a single dose of 20 mg/kg gabapentin administered orally to normal cats are summarized in Table 1 and the drug concentration curve is illustrated in Figure 1.

**Table 1.** Pharmacokinetic (PK) parameters of gabapentin in normal cats (single dose 20 mg/kg PO)

PK	Median (range)
C_max_ (ng/ml/mg)	242.0 (183.6–271.0)
T_max_ (h)	1.5 (1–2)
AUC (h*ng/ml/mg)	1587 (1449–1723)
K_el_ (1/h)	0.17 (0.14–0.2)
Half-life*	4.1 ± 0.5

* Half-life values are reported as harmonic mean with jackknife pseudo-SD

C_max_ = maximum serum concentration; T_max_ = time to maximum serum concentration; AUC = area under the curve; K_el_ = elimination rate constant

### Limited sampling study

**Figure 2:**
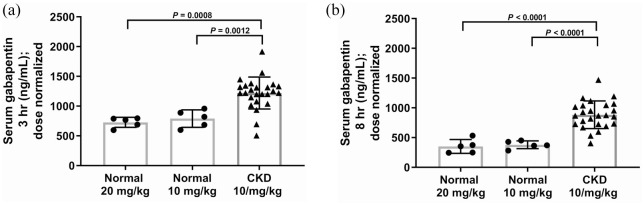
(a) Three hour and (b) 8 h post-administration dose-normalized gabapentin serum concentrations in cats with chronic kidney disease (CKD [10 mg/kg; n = 25]) and normal cats (20 mg/kg and 10 mg/kg; n = 5). Box and whiskers represents mean ± SD, and symbols are individual observations. Cats with CKD had significantly higher dose-normalized gabapentin serum concentrations than normal cats at either dose at both time points

**Figure 3:**
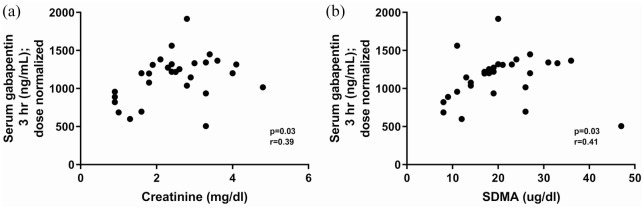
Correlation between 3 h serum dose-normalized gabapentin concentration and (a) serum creatinine concentration and (b) serum symmetric dimethylarginine (SDMA) concentration in five normal cats and 25 cats with chronic kidney disease that received a single 10 mg/kg PO dose of gabapentin

**Figure 4:**
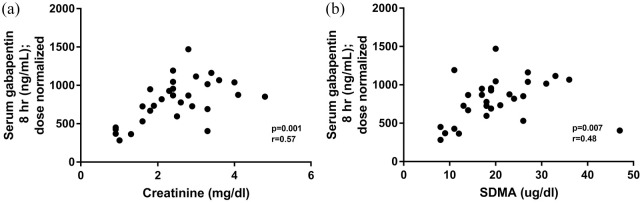
Correlation between 8 h serum dose-normalized gabapentin concentration and (a) serum creatinine concentration and (b) serum symmetric dimethylarginine (SDMA) concentration in five normal cats and 25 cats with chronic kidney disease that received a single 10 mg/kg PO dose of gabapentin

Dose-normalized gabapentin serum concentrations at 3 h and 8 h post-administration for normal cats (20 mg/kg and 10 mg/kg) and cats with CKD (10 mg/kg) are illustrated in Figure 2. Cats with CKD had significantly higher dose-normalized serum gabapentin concentrations than normal cats (for both doses) at both time points. At both the 3 h and 8 h time points, 92% of CKD cats that received 10 mg/kg had a dose-normalized serum concentration higher than the upper range of normal cats that received 20 mg/kg. Dose-normalized gabapentin serum concentrations at 3 h were significantly correlated with serum creatinine (Figure 3a) and SDMA (Figure 3b). Dose-normalized gabapentin serum concentrations at 8 h were also significantly correlated with serum creatinine (Figure 4a) and SDMA (Figure 4b).

### Compliance scores

**Figure 5:**
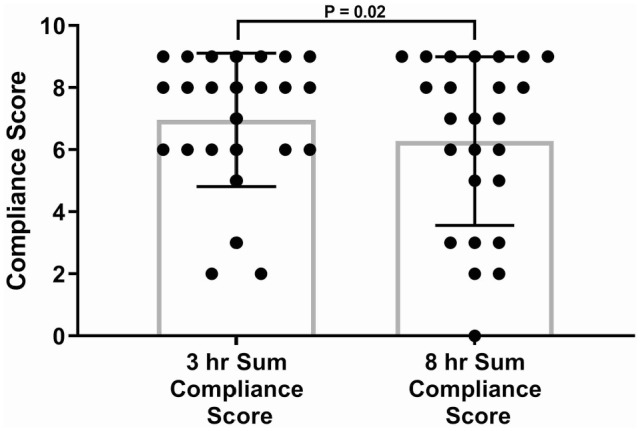
Compliance scores for cats with chronic kidney disease (CKD; n = 25) 3 h and 8 h after administration of a single 10 mg/kg PO dose of gabapentin (*P* = 0.02). CKD cats were more compliant at 3 h. Box and whiskers represent mean ± SD and symbols are individual observations

**Figure 6:**
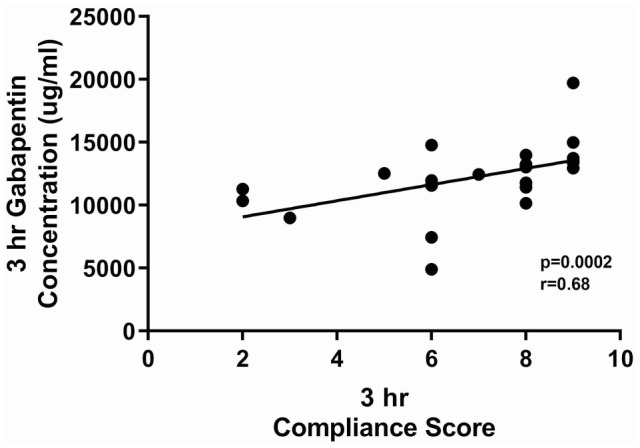
Correlation between compliance scores and gabapentin serum concentrations 3 h after cats with chronic kidney disease (n = 25) received a single 10 mg/kg PO dose of gabapentin. Cats with higher serum concentrations were more compliant. Box and whiskers represent mean ± SD and symbols are individual observations

Compliance scores for cats with CKD were significantly increased at 3 h vs 8 h (*P* = 0.02; Figure 5). There was a significant correlation between 3 h serum gabapentin concentrations and compliance scores (cats with higher serum concentrations were more compliant) (*P* = 0.0002, *r* = 0.68 [Figure 6]). No CKD cats in this study were considered to be overly sedated at the 10 mg/kg dose.

## Discussion

This study demonstrated that cats with CKD had significantly higher dose-normalized gabapentin serum concentrations in comparison with clinically normal cats. Furthermore, the serum concentration of gabapentin was positively correlated with both serum creatinine and SDMA at both 3 h and 8 h after administration indicating that cats with later stages of disease may be more affected. These findings are not surprising given that gabapentin undergoes 100% renal elimination in humans, and based on the results of this study, similar drug disposition may exist in cats.^7^ An increase in volume of distribution could result in a similar effect; however, this study is preliminary in nature and was not designed to fully elucidate which factors (absorption, distribution or clearance) would result in increased serum concentrations in cats with CKD. Nonetheless, clinicians should be aware that increased serum concentrations of gabapentin occur in cats with CKD, and similar to the recommendations in human medicine, a dose decrease is likely needed.^7,8^ These findings may also have implications for repeated dosing and/or chronic use (eg, management of osteoarthritis), and additional studies are needed to determine best dosing recommendations in this subset of patients.

It would have been helpful to assess if there is an age effect on gabapentin PK in cats, in addition to the effect of CKD. A limitation of the current study was that the clinically normal cats enrolled were all relatively young, compared with the cats with CKD, and a normal geriatric group, or age matching was not possible. However, in humans, it has been determined that the majority of the alteration in gabapentin PK that occurs with age is, in fact, attributable to decreased renal function in this age group, and once data are adjusted for glomerular filtration rate, gabapentin clearance was not affected by age.^11^ It is unclear whether a similar conclusion can be drawn in cats.

Two cats with CKD were excluded from data analysis because their serum gabapentin concentrations were higher at 8 h than at 3 h (verified with repeat analysis). It is unknown whether this phenomenon is actually related to a PK effect, or might instead be related to a delay in capsule transit or dissolution in the stomach. The two cats for which this occurred were not markedly more azotemic than other cats participating in the study (serum creatinine concentrations were 2.6 mg/dl and 3.2 mg/dl, respectively). Therefore, it is challenging to infer any definitive conclusions from this observation.

Patient compliance was found to be correlated to serum gabapentin concentrations at 3 h after administration, and also to be decreased at the 8 h assessment. These observations suggest a relationship between serum concentrations and patient compliance. Compliance scoring could not all be performed by the same individual, which could have introduced variability. No baseline compliance scores were performed, which precluded assessment of the degree of compliance, in comparison to normal. However, the aim of this study was centered on the altered serum concentrations of gabapentin in CKD, and the compliance scores were performed as a secondary aim to provide a clinical correlate with serum concentrations. Additionally, no comparison in compliance scores were made between normal and cats with CKD, as that was not the aim of the study; it was also not the aim of the study to assess compliance in normal cats at the 10 mg/kg dose.

Serum concentrations of gabapentin at an oral dose commonly used for stress reduction during veterinary visits (20 mg/kg) were found to closely match what would be predicted based on non-parametric superposition of previously published results for dosing at 10 mg/kg, indicating that the drug displays dose proportionality in healthy cats within this dose range.^9,10^ This is also supported by the dose-normalized 3 h and 8 h concentrations in the current study. The original design of the study included compliance scores for the clinically healthy cats during the full PK study, but this procedure was found to be confounded by the presence of jugular catheters and e-collars necessary for the PK study and thus was not performed.

Owing to the design of this research study (the desire to compound doses in 5 mg increments), it was the professional opinion of the pharmacist that it would be best to compound with bulk ingredients to achieve the needed strengths per capsule as the US Food and Drug Administration (FDA) allows manufacturers to make products that fall within a 10% error range, and this could amplify error. Although the FDA draft guidance on compounding animal drugs from bulk substances (GFI #256) allows a pharmacist to compound substances from bulk ingredients in certain situations, it should be noted that gabapentin is not listed as an accepted bulk chemical ingredient therein. However, the pharmacist followed compounding standards set by USP chapter 795 in the preparation of the capsules for this research study. To best follow federal laws and regulations, the authors encourage the use of FDA-labeled, manufactured products in compounded medications to help ensure accuracy and safety of veterinary compounded products in clinical practice.

## Conclusions

Dose reduction is likely needed when prescribing gabapentin to CKD patients as dose-normalized serum gabapentin concentrations were significantly higher in the majority of cats with CKD in this study than in normal cats. Additionally, dose-normalized serum gabapentin concentrations were positively correlated with serum creatinine indicating that greater caution is needed with advancing disease stage.
